# Effects of different doses of complete Freund’s adjuvant on nociceptive behaviour and inflammatory parameters in polyarthritic rat model mimicking rheumatoid arthritis

**DOI:** 10.1371/journal.pone.0260423

**Published:** 2021-12-08

**Authors:** Ain’ Sabreena Mohd Noh, Tan Dai Chuan, Nurul Ajilah Mohamed Khir, Anani Aila Mat Zin, Anis Kausar Ghazali, Idris Long, Che Badariah Ab Aziz, Che Aishah Nazariah Ismail

**Affiliations:** 1 Department of Physiology, School of Medical Sciences, Universiti Sains Malaysia Health Campus, Kelantan, Malaysia; 2 Department of Chemistry, Faculty of Science, Universiti Putra Malaysia, Selangor, Malaysia; 3 International Medical School, Management and Science University, Selangor, Malaysia; 4 Department of Pathology, School of Medical Sciences, Universiti Sains Malaysia Health Campus, Kelantan, Malaysia; 5 Biostatistics and Research Methodology Unit, School of Medical Sciences, Universiti Sains Malaysia Health Campus, Kelantan, Malaysia; 6 Biomedicine Program, School of Health Sciences, Universiti Sains Malaysia Health Campus, Kelantan, Malaysia; 7 Brain and Behaviour Cluster, School of Medical Sciences, Universiti Sains Malaysia Health Campus, Kelantan, Malaysia; University of Messina, ITALY

## Abstract

Complete Freund’s adjuvant (CFA) has been used to develop the arthritic or inflammatory condition in the animal, but there is a lack of information concerning high CFA doses on nociceptive behaviour and inflammatory parameters. This study aimed to compare the effects of different high doses of CFA in rat to closely mimic nociceptive and inflammatory parameters of rheumatoid arthritis (RA) in humans. Twenty-four male Sprague-Dawley rats were randomly divided into four groups (n = 6): Control (C), CFA-induced polyarthritic groups at 5.0 mg/mL (CFA 5.0), 7.5 mg/mL (CFA 7.5) and 10.0mg/mL (CFA 10.0). The rats’ right hindpaw was inoculated with CFA intradermally and developed into a polyarthritic state within 20 days. Nociceptive behavioural assessments, including von Frey and hot plate tests and spontaneous activities, were conducted on day 0, 7, 15 and 20. Bilateral ankle joints diameter and circumference, full blood count, joints and paw histological examinations were also conducted throughout the study period. Based on the results, CFA 5.0 and CFA 7.5 groups showed a significant increase in spontaneous activities and development of thermal hyperalgesia but no change in body weight and food intake, no development of tactile allodynia and haematological indices, and no significant morphological changes of joints histology. Meanwhile, CFA 10.0 group demonstrated significant and constant changes in all nociceptive and inflammatory parameters investigated. In conclusion, CFA at the dose of 10mg/mL has the most potential and reliable dosage to develop polyarthritis in a rat model to mimic RA condition in humans.

## Background

RA is an autoimmune disease in which synovila cells, cartilage and bone are attacked by the pathologic immune responses resulting in joint damage and permanent disability [1]. In a survey conducted by the United States Health Interview Survey from 2013 to 2015, it was reported that the annual prevalence of doctor-diagnosed arthritis was 22.7% [2]. The occurrence of RA is reported to be more common in women than men, especially before menopause [3]. Ollier et al. [4] suggested that men are protected from RA to a certain extent due to hormonal factors and a strong genetic component for the disease development. RA is also defined as dysregulated inflammatory processes in the joint synovium, destroying the joint cartilaginous and bony elements, resulting in pain and disability [5]. The RA-associated pain, exhaustion and joints impairment contribute to a major fall in health-related quality of life. The exact cause of RA remains unclear, but some factors may increase the risk of disease development. Therefore, lowering the prevalence and impact of arthritis is critical to plan future clinical and public health needs [6].

Adjuvant-induced arthritis (AIA) animal model has been established for decades to study the pathogenesis of arthritis, including rheumatoid arthritis (RA), gout and osteoarthritis, and to evaluate the effectiveness of certain anti-arthritic drugs [7, 8]. According to Jeffery [9], the RA models are relatively easy to execute, have good data reproducibility and generally short-lived. There are several types of adjuvants suggested including complete Freund’s adjuvant (CFA), incomplete Freund’s adjuvant (IFA), collagen and carrageenan [10, 11]. In order to mimic certain disease, several important selection criteria should be considered such as morphological similarities to present as in humans [11]. The animal models should have comparable clinical features, including acute-to-enhanced inflammatory reactions at the joints and periosteal or endosteal of the bone [12, 13] as well as pain such as allodynia, hyperalgesia and spontaneous pain [14].

The administration of CFA has been known to cause a series of inflammatory reactions. The injection of CFA at the rat’s footpad results in the cutaneous inflammation presented as reddish and swollen [15, 16] beginning as early as 2 hours and peaks within 6 to 8 hours [17]. This acute inflammation occurs for 3–4 days [15] in which the levels of erythrocyte sedimentation rate (ESR), blood neutrophil and leukocyte counts start to increase on the fourth day post-CFA injection [18, 19]. It is then followed by the development of hyperalgesia and oedema in ankle and dorsal region of tarsus at 1–2 weeks post-CFA injection [17, 20] due to the extensive infiltration of neutrophil and proliferation of synovial lining [21]. The inflammation starts to affect the adjacent joints [21] as macrophages release pro-inflammatory cytokines such as tumor necrosis factor-α (TNF-α) interleukin-1β (IL-1β). TNF-α may work together with IL-1β to induce intracellular adhesion and migration, production of acute-phase proteins, proteolytic enzymes and angiogenesis [19, 22], monocyte-chemoattractant protein-1 (MCP-1), macrophage-inflammatory protein-1α (MIP-1α), interleukins (i.e. IL-6 and IL-12) and epithelial–neutrophil activating peptide-78 (ENA-78) in the affected joint synoviocytes [23–25]. The devastating inflammatory reactions eventually result in chronic arthritis due to positive feedback mechanisms [21, 26].

Amongst the commercially-available adjuvants, CFA is the most commonly opted by the researchers to develop arthritic rat model [27, 28] or to be used as a vehicle for vaccination in animals [29, 30]. The early proposed method on developing arthritic model was via intradermal injection of CFA at the base of the rat’s tail which yields prolonged lapsing-remitting arthritis involving several joints [20, 31, 32]. However, due to the multiple disadvantages of this administration route that resulted in severe arthritis, it was progressively modified to reduce the severity via local administration of CFA either into or around the tibiotarsal joint of the rodents (i.e development of monoarthritis models) [33–35]. In order to standardise the arthritic progression in rodents as indicated in human, Costa et al. [7] categorises the disease progression into several phases: 1) pre-clinical (1–10 days), 2) acute (15–30 days), 3) post-acute (30–50 days) and 4) late (more than 50 days). In fact, several approaches of administering CFA were introduced to develop AIA model in the previous literature either single or double inoculations which may involve one or two locations of CFA inoculations. The single administration of CFA requires higher dosage ranging from 300 μg to 20 mg/mL to ensure the prolonged occurrence of arthritic complications such as joints oedema with chronic hyperalgesia [15, 22, 33, 36–39]. However, the remission of CFA inoculation at a lower dosage is also suggested by a number of studies to minimize the arthritic complications such as inoculation of various dosage of CFA at the right food pad and base of the rat’s tail [16] or 0.5–1.0 mg/mL of CFA at tibiotarsal or tibiofemoral joints [40]. Nonetheless, the different number of CFA inoculations may bring different progression of disease development as the use of lower CFA dosage is not sufficient to cause arthritic pain unless the second CFA inoculation is administered to the rat [40]. Although several approaches and dosage of CFA are suggested in the previous literature, the severity of arthritic complications experienced by the rat associated with CFA dosage seems to be less attentive and focused. It is actually important if the lower dosage of CFA that produce the similar presentation of arthritic complications in terms of inflammation and pain could be produced. Moreover, not a single study has looked at the effects of different CFA doses in inducing chronic polyarthritic condition mimicking RA in terms of nociceptive behaviours and inflammatory parameters in rat. Therefore, the purpose of this study was to compare the effects of CFA at different doses on nociceptive behaviour and inflammatory markers in the polyarthritis rat model. This study is also aimed to identify the optimal single dose of CFA to produce both inflammatory and nociceptive effects mimicking RA in human. It is hypothesised that different dosages of CFA produce dose-dependent effects on the nociceptive behaviours and inflammatory parameters in rats.

## Methodology

### Materials

CFA was purchased from Sigma-Aldrich (USA) ready-to-use, pale yellow liquid. Each 10 ml CFA vial contained 10 mg heat-killed, dried *Mycobacterium butyricum* (strain H37Ra), 1.5 ml mannide monooleate and 8.5 ml paraffin oil. The CFA liquid can sometimes appear cloudy since it contains particulate matter.

### Animals

Twenty-four male Sprague-Dawley rats (270–300 g, 8–10 weeks old) were housed individually in the animal room with a constant temperature of 22 ± 1.0 °C and a 12-hour alternating light-dark cycle. The sample size for each group of the animals (n = 6) was identified by using G* power version 3.0.10 software with α (type-1 error) of 0.05, power of 0.8 and effect size of 0.85 (expert opinion) and 20% drop-out. The rats were fed with the standard food pellets and water *ad libitum*. Then, they were randomly divided into four groups (n = 6): (a) Control (C), and CFA-induced chronic polyarthritic groups at different doses (b) 5.0 mg/mL (CFA 5.0), (b) 7.5 mg/mL (CFA 7.5) and (c) 10.0mg/mL (CFA 10.0). The protocols of this study were designed to minimise animal suffering has been approved by USM Institutional Animal Care and Use Committee, Universiti Sains Malaysia, Malaysia (USM IACUC) [USM/IACUC/2019/ (116) (984)].

### Induction of arthritis

Complete Freund’s adjuvant containing attenuated *M*. *butyricum* was dissolved in mineral oil as a vehicle [15, 33, 41], and prepared in three doses: 5.0 mg/mL (low dose), 7.5 mg/mL (moderate dose) and 10.0 mg/mL (high dose). The arthritis induction method was adapted from Nasuti et al. [15] by injecting 100 μL of CFA intradermally at the right hind metatarsal footpad under isoflurane anaesthetisation. The control (C) group was injected with the sterile vehicle at the same site.

### Change of body weight and total food intake

The rats’ body weight was measured on day 0, 3, 6, 7, 9, 15 and 20 and expressed as the percentage of change in body weight relative to the baseline weight (day 0) as shown in the formula:

$$\%\;\text{Change}\;\text{of}\;\text{body}\;\text{weight}=\frac{\text{BW}\;\text{on}\;\text{Day}\;N-\text{BW}\;\text{on}\;\text{Day}\;0}{\text{BW}\;\text{on}\;\text{Day}\;0}\times 100\%$$

in which ‘N’ refers to the specific day when the body weight was recorded. Meanwhile, the total food intake of the rats in each group was recorded for 20 days of experimentation.

### Pain behaviour assessments

The behavioural assessments consisted of spontaneous activities, thermal hyperalgesia and tactile allodynia. These tests were performed on day 0 (baseline), 7 and 20 due to the trend of changes in joint inflammation on the specified days in the CFA-induced chronic arthritic models as demonstrated by previous studies [7, 33, 42]. In addition, the evaluation of the pain behaviours was performed by two persons who were blinded to the treatment groups to avoid inter-observer bias [33].

### Spontaneous activities assessment

The rats were placed separately in a Plexiglas chamber and allowed to acclimatise for 15 minutes. The standing and walking paw pressures representing the spontaneous activities adapting from Coderre and Wall [43], Butler et al. [33] and Neugebauer et al. [18] were recorded for 5 minutes.

#### Standing paw pressure

In this test, the amount of weight (paw pressure) the rat was willing to place on its inoculated hind paw was measured and graded according to the scores adapted from Coderre and Wall [43] and Butler et al. [33] as the following:

0 = normal paw pressure, equal weight on both hind paws.
1 = slightly reduce paw pressure, the paw is completely on the floor, but toes are not spread.
2 = moderately reduce paw pressure, foot curls with only some parts of the foot lightly touching the floor.
3 = severely reduce paw pressure; foot elevates completely.

The higher score represents the increased severity of the paw pressure experienced by the animal.

#### Walking paw pressure

In this assessment, the extent of the limp or gait in rats altered by the CFA inoculation was evaluated. The scores for walking paw pressure assessment was based on Coderre and Wall [43] and Butler et al. [33] as follows:

0 = Normal gait.
1 = Slight limp, visible over-flexion of the injected limb.
2 = Moderate limp, the paw of injected hind limb only briefly touches the floor.
3 = Severe limp, three-legged gait.

The higher score indicates more severe walking paw pressure experienced by the animals.

### Tactile allodynia

Tactile allodynia assessment was conducted on the rat following Naeini et al. [41] and Zulazmi et al. [44]. The assessment was conducted in a quiet room to prevent any environmental-induced emotional disruption. First, the rats were placed on the wire mesh floor separated by compartments and acclimatised 15 minutes before the experiment. Then, the paw withdrawal threshold was measured by applying the von Frey microfilament with a gradual increase in force to the mid-plantar surface of the hind paw. The pressure that evoked paw withdrawal represented maximal noxious threshold and expressed in gram [45]. The procedure was carried out on the CFA-inoculated hind paw (ipsilateral hind paw) of the rat, followed by the CFA non-inoculated hind paw (contralateral hind paw). The stimulus was given three times, and the mean value of the paw withdrawal threshold was measured and recorded [44]. A 10-minute interval was allocated between mechanical stimulations to prevent sensitisation and adaptation to the stimuli [46].

### Thermal hyperalgesia

Thermal hyperalgesia in the rats was evaluated using a hot plate analgesia meter immediately after the von Frey test. The animals were acclimatised to the examination room for 10 minutes prior to test initiation. Several indicators were used to denote the animal’s thermal threshold: the hind paw lick, back paw flick, or hop from the onset of the thermal stimulation. First, the hot plate surface is continuously heated at 52.5 °C [47, 48], and a cut-off time of 12 s was set to prevent any tissue damage in the absence of response [49]. The animal was immediately removed from the hot plate analgesia meter after the test.

### Sacrifice of the animals

On day 20, the rats were sacrificed by an overdose of sodium pentobarbitone intraperitoneally. The rat’s bilateral ankle joints and the intraplantar surface of the injected hind paws were collected for histology purpose.

### Inflammatory parameters in the ankle joint and paw skin of the rat

#### Measurement of ankle joint circumference

Both ankle joints circumference and diameter (representing oedema formation) were measured at several time points: day 0 (adjuvant induction, baseline value), 3, 6, 7, 9, 15 and 20 to evaluate the trend of changes in inflammatory conditions at the affected joints [41, 50, 51]. Each ankle joint (hind paw) was measured by using a digital calliper (Duratool, China) for diameter (cm) and thread for circumference (cm) measurements to assess the development of chronic inflammation. First, the circumference of the joint was measured by looping the thread around the ankle joints and gently tightened. Then, the thread was carefully removed and measured using a metric ruler. According to Costa et al. [7] the occurrence of oedema, which marks the development of chronic arthritis in the ankle joint of the rats, begins to develop on day 7 and decreases slowly after day 20 following the CFA injection.

### Histology procedure for hind paw processing

Bilateral ankle joints and the intraplantar surface of the inoculated (ipsilateral) hind paw of the rats were dissected and post-fixed in 4% formalin [50]. Only the joints were then placed in a decalcifying solution (3% nitric acid solution) [51, 52] for 24–72 hours [50]. After that, both tissues were embedded in paraffin, sectioned at 4 μm, stained with haematoxylin and eosin (H&E) staining and observed under a light microscope connected to an image analyser. Then, the qualitative assessment for both tissues were compared between groups to identify the presence of inflammatory cells (for hind paw and joint tissues), synovial hyperplasia and any alteration of the joint space (for the joint tissues) based on Cui et al. [53] and Khader et al. [54].

### Blood collection

Following the termination of the rat on day 20, a midline incision was made, and the blood sample was taken from the rat’s heart via cardiac puncture. Blood samples (3 mL) were stored in ethylenediaminetetraacetic acid (EDTA) tubes, centrifuged (2000 × g, 5 min) at 4 °C [55] and subjected to a full blood count.

### Statistical analysis

Repeated measures analysis of variance (ANOVA) was employed to analyse spontaneous activities (i.e. walking and standing paw pressures), tactile allodynia, thermal hyperalgesia and ankle joints measurements with posthoc Bonferroni test. Meanwhile, one-way ANOVA was utilised to analyse body weight changes, total food intake, and haematological analysis with post hoc Scheffe’s test. All data were expressed as mean ± standard error of the mean (SEM) and analysed using the Statistical Package for the Social Sciences (SPSS) version 21 software (IBM, United States). A value of *p* < 0.05 was considered statistically significant.

## Results

### Reduction of body weight in polyarthritic rats

**Fig 1:**
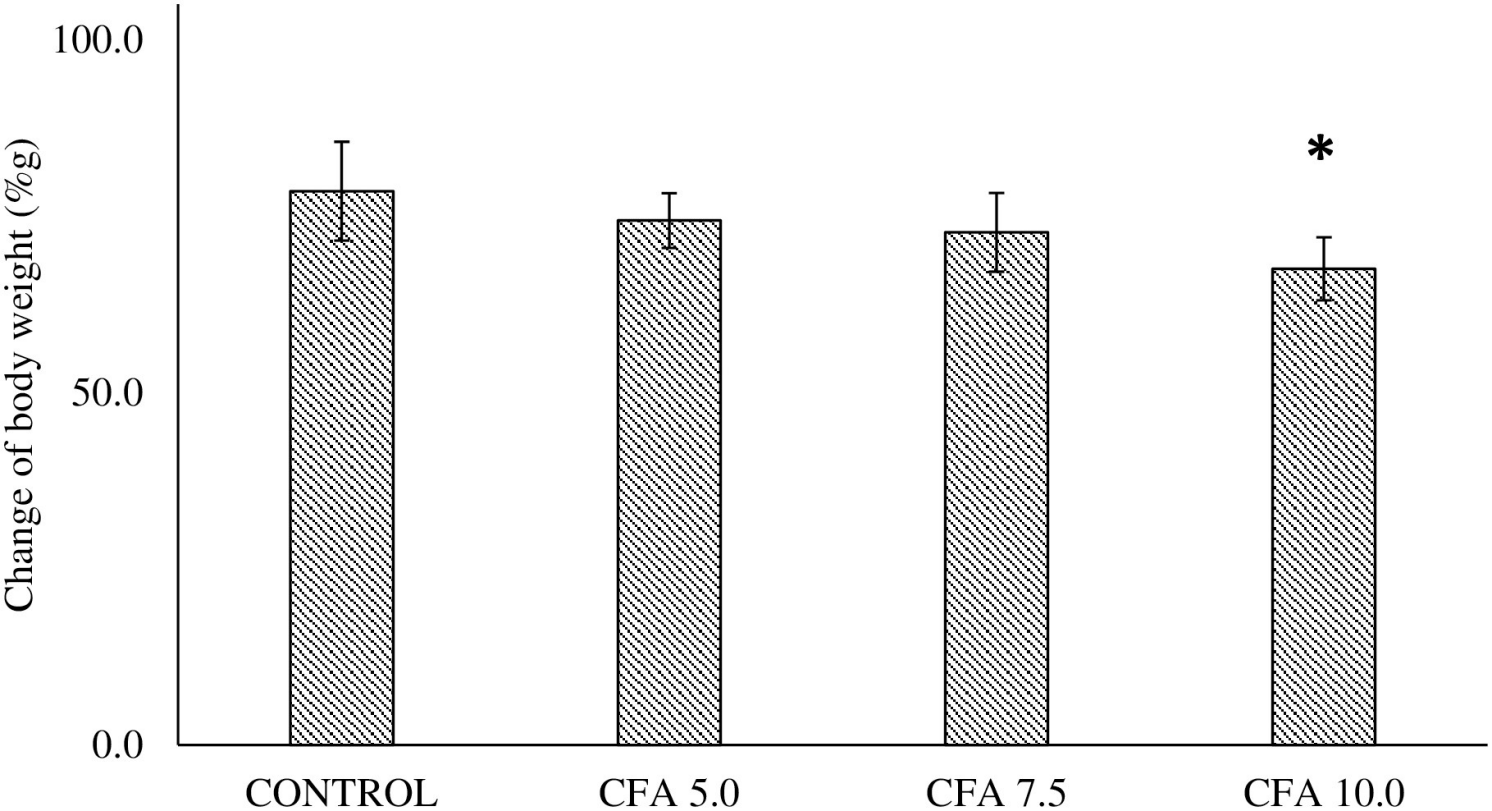
Percentage of body weight changes in CFA treatment groups (n = 6). The data are presented as mean ± standard error of the mean (SEM). *p < 0.05 statistical comparison between the group receiving CFA injection at 10 mg/mL to the control group.

There was a statistically significant difference between the groups as determined by one-way ANOVA (F_3,20_ = 4.318, p < 0.05) (Fig 1). Post-hoc Scheffe’s test revealed that the body weight was significantly reduced following the arthritic induction in CFA 10 group (p < 0.05, 95% CI -1.556 to -20.444) compared to control group with no significant different in other CFA groups. No significant difference was detected between the CFA 5.0 and CFA 7.5 groups compared to control group (p > 0.05) (Fig 1).

### No change of total food intake in polyarthritic rats

**Fig 2:**
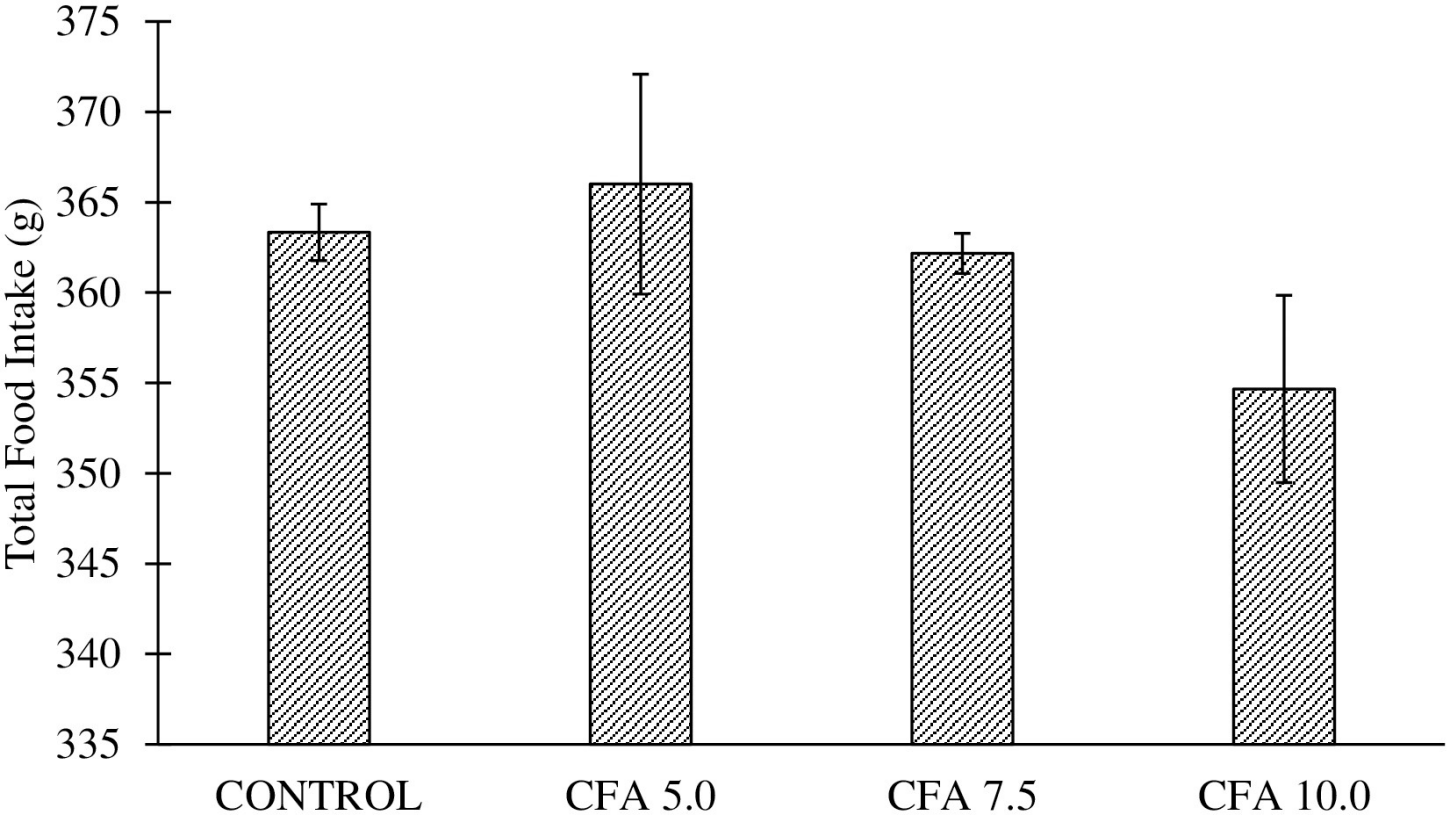
Total food intake in the groups for 20 days of experimentation. No significant changes were detected between the groups.

There was no significant difference in the total food intake across the groups as reported by one-way ANOVA (F_3,20_ = 1.397, p > 0.06) (Fig 2).

### Reduced spontaneous activities in chronic polyarthritic rats

**Fig 3:**
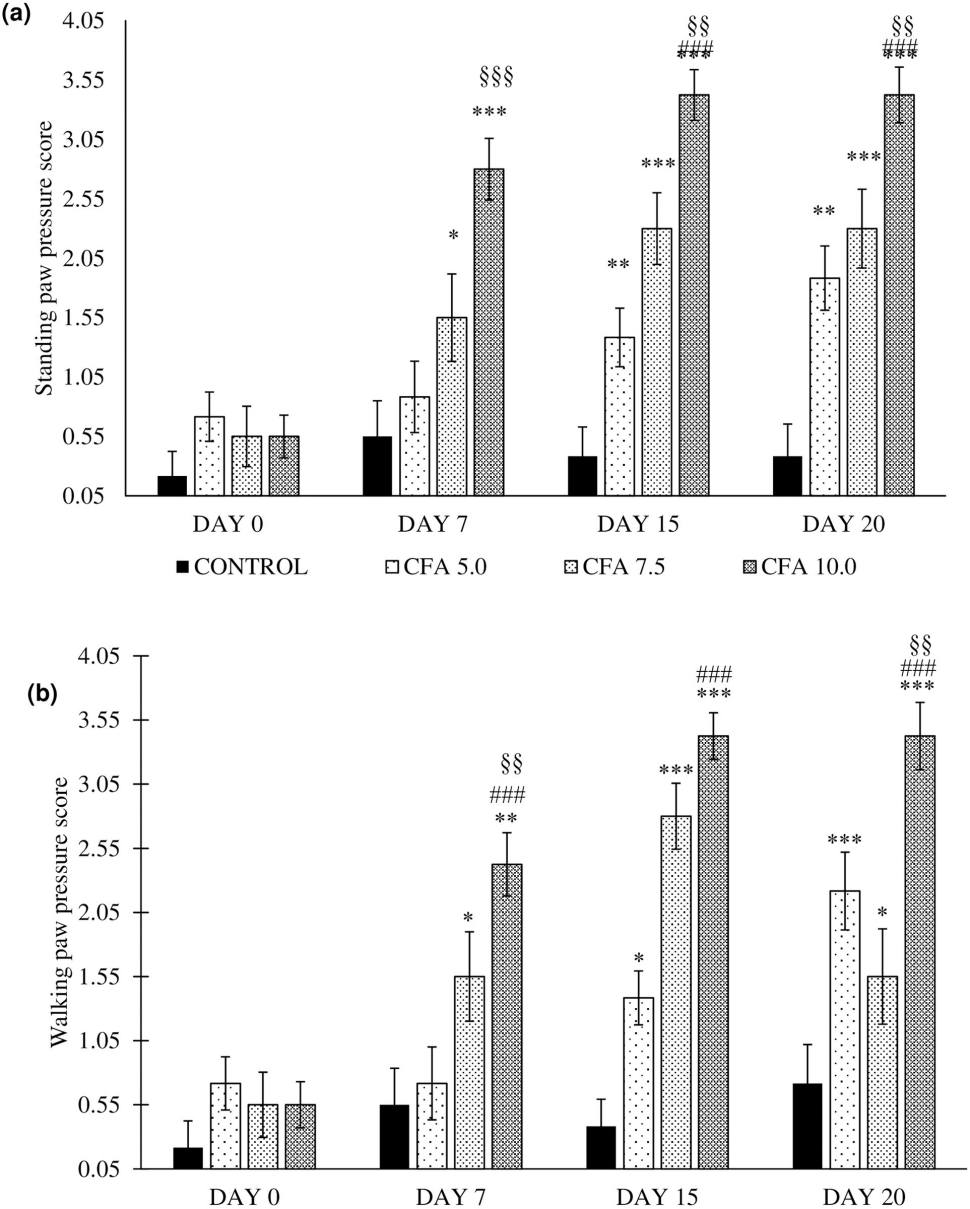
Spontaneous activities as indicated by (a) standing paw pressure and (b) walking paw pressure in the groups on day 0, 7, 15 and 20 (n = 6). The data are presented as mean ± standard error of the mean (SEM). *p < 0.05, **p < 0.01, ***p < 0.001 Statistical comparison to control group, ^###^p < 0.001 Significant comparison to CFA 5.0 group and ^§§^p < 0.05, ^§§§^p < 0.001 Significant comparison to CFA 7.5 group. The CFA 10 group showed the most consistent and the highest score for both standing and walking paw pressure compared to the control group, especially on day 7, 15 and 20.

The standing paw pressure revealed the significant main effects of time (F_3,20_ = 12.7, p < 0.001), group (F_3,20_ = 43.2, p < 0.001), and a significant group by time interaction (F_3,20_ = 34.6, p < 0.001). The post-hoc Bonferroni test reported significant increase in the standing paw pressure in CFA 5.0 (p < 0.01, 95% CI 0.269 to 1.398), CFA 7.5 (p < 0.001, 95% CI 0.661 to 1.923) and CFA 10.0 (p < 0.001, 95% CI 1.639 to 2.694) compared to control group. There was no significant comparison in the standing paw pressure between CFA 5.0 and CFA 7.5 groups. However, the CFA 10.0 group showed the significant and constant increase of the standing paw pressure scoring compared to CFA 5.0 (p < 0.001, 95% CI 0.806 to 1.861) and CFA 7.5 (p < 0.01, 95% CI 0.277 to 1.473) (Fig 3a).

Meanwhile, the results of walking paw pressure demonstrated a significant difference of time (F_3,20_ = 12.7, p < 0.001), group (F_3,20_ = 43.2, p < 0.001), and a significant group by time interaction (F_3,20_ = 34.6, p < 0.001). Post-hoc Bonferroni test revealed a significant increase in standing paw pressure in between CFA 5.0 (p < 0.01, 95% CI 0.234 to 1.349), CFA 7.5 (p < 0.001, 95% CI 0.523 to 1.769) and CFA 10.0 (p < 0.001, 95% CI 1.468 to 2.511) compared to control group. The higher scoring for walking paw pressure was demonstrated by CFA 10.0 group than the CFA 5.0 (p < 0.001, 95% CI 0.677 to 1.7192) and CFA 7.5 (p < 0.01, 95% CI 0.253 to 1.435) groups. There was no significant difference in walking paw pressure scoring between CFA 5.0 and CFA 7.5 groups (Fig 3b).

According to the standing and walking paw pressure results, these remark the constant occurrence of abnormal spontaneous activities in CFA 10.0 group especially on day 7, 15 and 20 without fluctuations as shown in the CFA 5.0 and CFA 7.5 groups indicating the optimal dose of CFA to develop chronic polyarthritis mimicking RA in rat.

### Development of tactile allodynia in chronic polyarthritic rats

**Fig 4:**
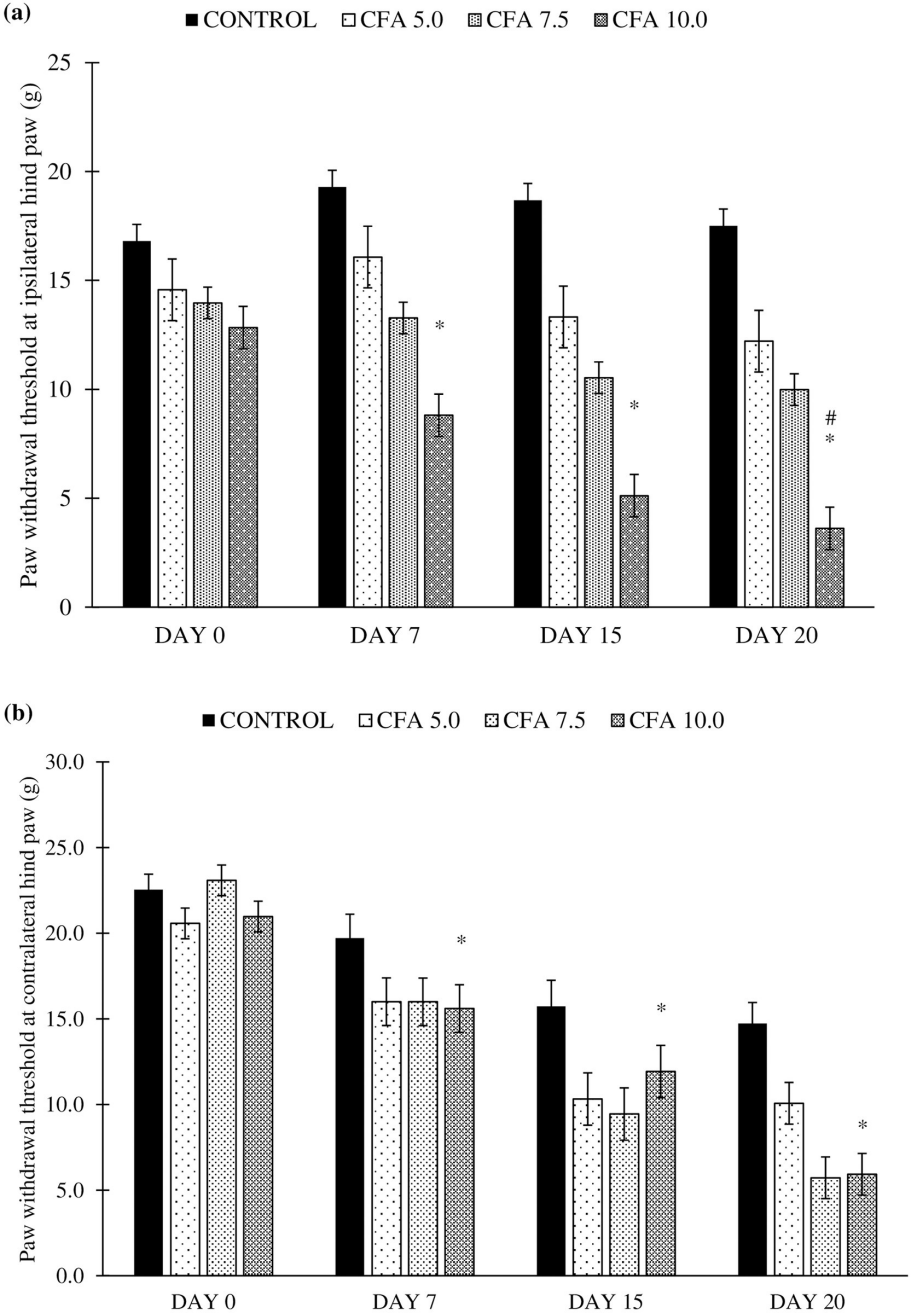
Paw withdrawal threshold at the (a) ipsilateral (CFA-inoculated) hind paw and (b) contralateral (non-CFA-inoculated) hind paw in the treatment groups on day 0, 7, 15 and 20 (n = 6). The values are presented as mean ± standard error of the mean (SEM). *p < 0.05 Statistical comparison to control group and ^#^p < 0.05 Statistical comparison to CFA 7.5. CFA 10.0 group showed a consistent significant reduction in paw withdrawal threshold compared to the control group, indicating the development of tactile allodynia in the rat, beginning with the ipsilateral followed by contralateral hind paws.

It was revealed that there was a significant difference detected in the paw withdrawal threshold at the ipsilateral hind paw between the time (F_3,20_ = 45.8, p < 0.05), group (F_3,20_ = 6.5, p < 0.05), and a significant time by group interaction (F_3,20_ = 50.7, p < 0.001). The statistical analysis with post-hoc Bonferroni test reported the significant reduction in the paw withdrawal threshold in CFA 10.0 group compared to control group (p < 0.05, 95% CI -6.795 to -0.63) and CFA 5.0 group (p < 0.05, 95% CI -6.612 to -0.448) whilst CFA 5.0 and CFA 7.5 groups showed no significant difference in the paw withdrawal threshold compared to control group (Fig 4a).

Meanwhile, the significant difference was also detected in the paw withdrawal threshold at the contralateral hind paw between the time (F_3,21_ = 66.529, p < 0.001), group (F _3,20_ = 6.555, p < 0.05), and a significant time by group interaction (F_9,43_ = 3.197, p < 0.05). Post-hoc Bonferroni test also reported a significant increase in paw withdrawal threshold at the contralateral hind paw in CFA 10.0 group compared to control group (p < 0.05, 95% CI -6.795 to -0.63) and CFA 5.0 group (p < 0.05, 95% CFA -6.613 to -0.448) starting on day 7 to 20. Meanwhile, no difference was found in other CFA groups for this parameter (Fig 4b). These results indicate that the tactile allodynia was developed in the CFA 10.0 group affecting the non-injected (contralateral) hind paw of the rat.

### Development of thermal hyperalgesia in chronic polyarthritic rats

**Fig 5:**
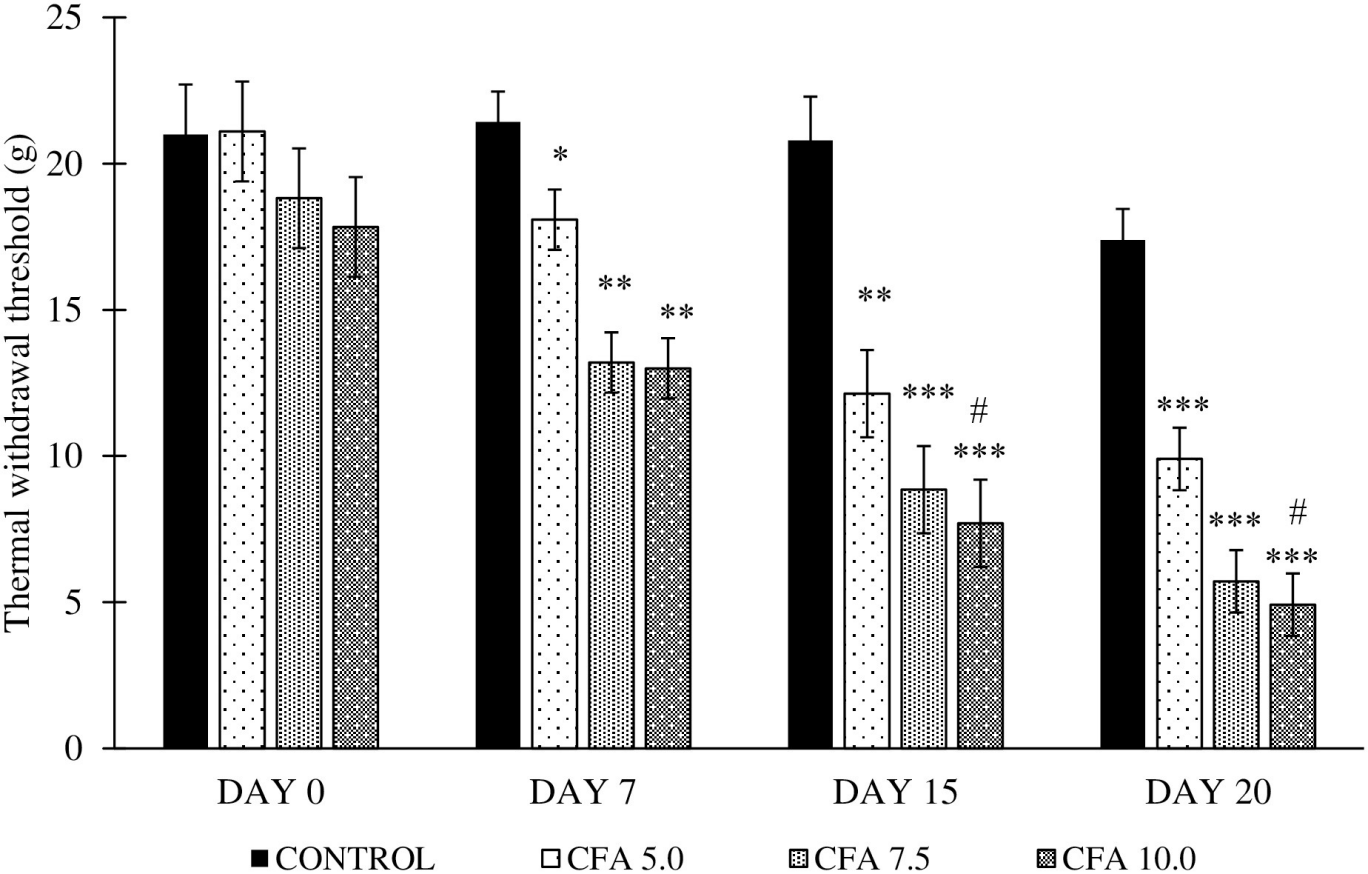
Thermal withdrawal threshold in the treatment groups on day 0, 7, 15 and 20 (n = 6). The values were expressed as mean ± standard error of the mean (SEM). *p < 0.05, **p < 0.01 and ***p < 0.001 Significant comparison to control group. #p < 0.05 Significant comparison to CFA 5.0 group. CFA 10.0 group demonstrated a significant gradual reduction in thermal withdrawal threshold, indicating the gradual development of thermal hyperalgesia throughout the experiment.

The analysis by one-way repeated measures ANOVA revealed a significant difference in thermal withdrawal threshold of polyarthritic-induced rats in time (F_3,20_ = 59.0, p < 0.001), group (F_3,20_ = 21.1, p < 0.001), and a significant time by group interaction (F_3,20_ = 5.3, p < 0.05). The post-hoc Bonferroni test reported a significant decrease in thermal withdrawal threshold between CFA 5.0 (p < 0.05, 95% CI 0.875 to 8.825) and CFA 7.5 (p < 0.001, 4.534 to 12.483) groups to control group. Amongst the CFA dosage, the rat inoculated with 10 mg/mL dose showed the significant gradual decrease in thermal withdrawal threshold compared to control (p < 0.001, 95% CI 5.317 to 13.266) and CFA 5.0 groups (p < 0.05, 0.425 to 8.416) especially on day 7 to 20 (Fig 5).

### Development of inflammatory oedema in ankle joints of chronic polyarthritic rats

#### Changes in ipsilateral joint circumference and diameter

**Fig 6:**
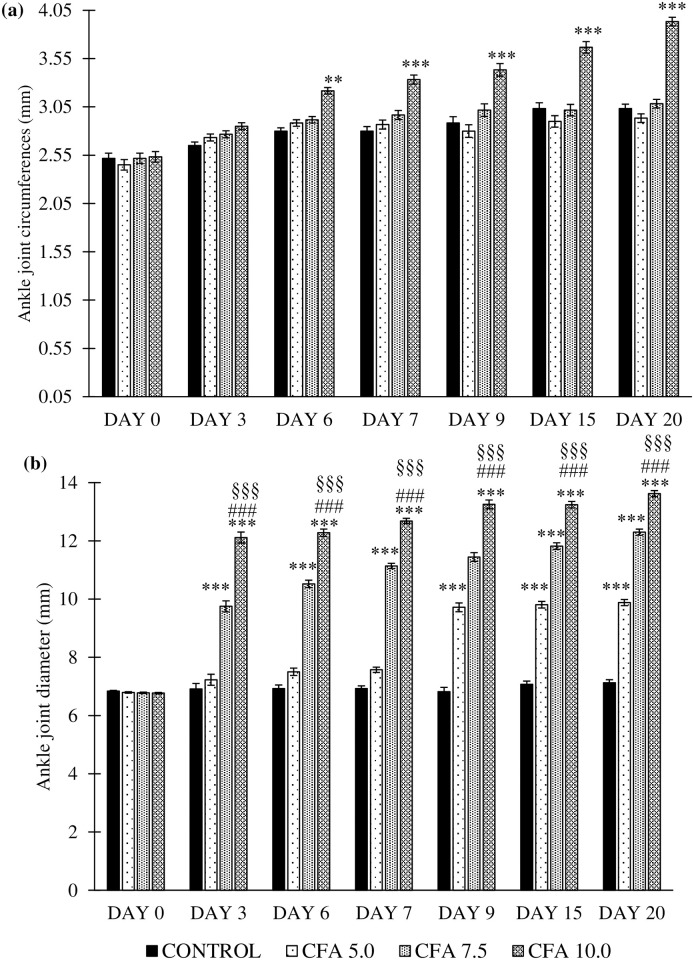
Changes in rats’ ankle joint (a) circumferences and (b) diameter at the ipsilateral (CFA-inoculated) hind paw in the treatment groups on day 0 to 20 (n = 6). The data are expressed as mean ± standard error of the mean (SEM). *p < 0.05 and **p < 0.001 significant comparison to control group. ^###^p < 0.001 significant comparison to CFA 5.0 group and ^§§§^p < 0.001 significant comparison to CFA 7.5 group. CFA 10.0 group demonstrated the most reliable and constant gradual increase in the ankle joint oedema at the ipsilateral side compared to other doses of CFA inoculation.

One-way repeated measures ANOVA revealed that there was a significant main effects in the time (F_6,18_ = 25.4, p < 0.001), group (F_3,20_ = 78.9, p < 0.001), and a significant group by time interaction (F_6,17_ = 41.8, p < 0.001) for the ankle joint circumference between the groups (Fig 5a). Post-hoc Bonferroni test revealed a significant increase in the ipsilateral ankle joint circumference of CFA 10.0 group compared to control (p < 0.001, 95% CI 0.574 to 0.354), CFA 5.0 (p < 0.001, 95% CI 0.596 to 0.376) and CFA 7.5 (p < 0.001, 95% CI 0.493 to 0.273) groups. No significant comparison was detected in the ankle joint circumference in CFA 5.0 and CFA 7.5 groups compared to control group (Fig 6a).

Meanwhile, the significant main effects were also reported for the ipsilateral ankle joints diameter by time (F_6,18_ = 54.0, p < 0.001), group (F_3,20_ = 685.1, p < 0.001), and a significant group by time interaction (F_5,18_ = 20733.2, p < 0.001) between the groups. Post-hoc Bonferroni test also demonstrated the significant increase of ankle joint diameter at the ipsilateral region between the CFA groups to control group (CFA 5.0: p < 0.001, 95% CI 1.174 to 1.112, CFA 7.5: p < 0.001, 95% CI 3.89 to 3.29 and CFA 10.0: p < 0.001, 95% CI: 5.349 to 4.75). The CFA 10.0 group demonstrated the highest increase of ankle joint diameter at the ipsilateral side within the time frame compared to the other CFA groups (p < 0.001) (Fig 6b).

#### Changes in contralateral ankle joint circumference and diameter

**Fig 7:**
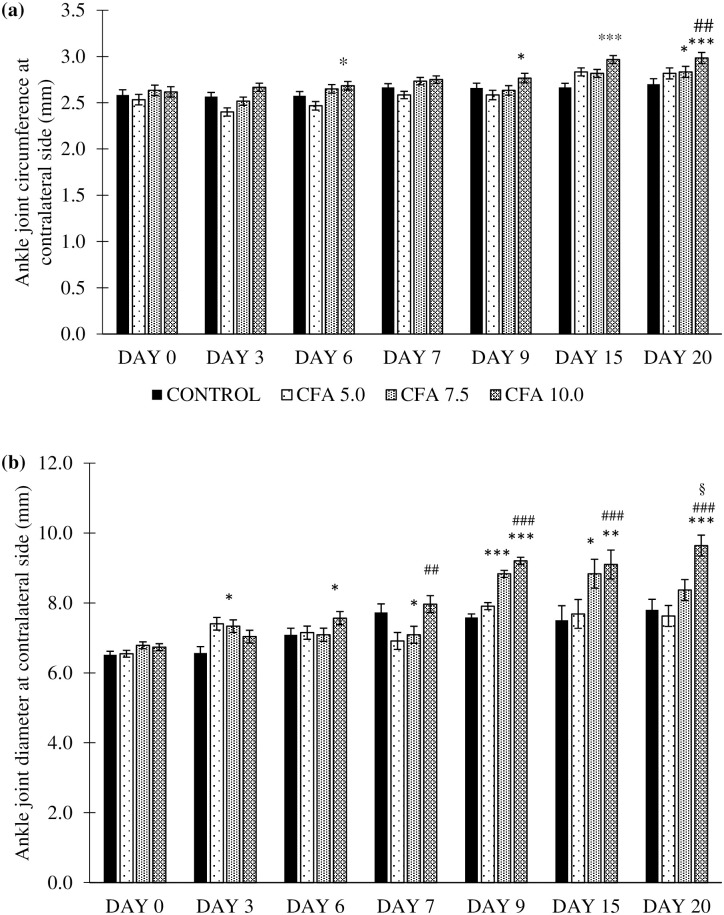
Ankle joint (a) circumference and (b) diameter at the contralateral (non-CFA- inoculated) hind paw in the treatment groups (n = 6). The values are presented as mean ± standard error of mean (SEM). *p < 0.05, **p < 0.01 and ***p < 0.001 significant comparison to control group. ^#^p < 0.05, ^##^p < 0.01 and ^###^p < 0.001 significant comparison to CFA 5.0 group. CFA 10.0 group showed a constant increase of inflammatory oedema at the contralateral (non-injected hind paw), indicating the development of chronic polyarthritis following CFA inoculation.

The results analysed by repeated measures ANOVA revealed significant differences in the contralateral ankle joint circumference by time (F_6,18_ = 21.355, p < 0.001), group (F_3,20_ = 5.609, p < 0.05), and a significant group by time interaction (F_5,18_ = 2.370, p < 0.05) between the groups. The post-hoc Bonferroni test reported a significant increase in the ankle joint circumference at the ipsilateral side in CFA 10.0 group compared to control group especially on day 6 to 20 (p < 0.05, 95% CI 0.011 to 0.279) and CFA 5.0 (p < 0.01, 95% CI 0.399 to 0.308). No significant difference was detected in the contralateral ankle joint circumference in CFA 5.0 and CFA 7.5 compared to control group (Fig 7a).

Meanwhile, there was also significant main effects in the time (F_6,18_ = 24.837, p < 0.001), group (F_3,20_ = 17.603, p < 0.001), and a significant group by time interaction (F_5,18_ = 42.912, p < 0.001) in the diameter of the contralateral ankle joint across the groups. The analysis by post-hoc Bonferroni showed that the significant increase in contralateral ankle joint diameter in CFA 7.5 and CFA 10.0 groups compared to control group especially on day 9, 15 and 20 (p < 0.05, 95% CI 0.081 to 0.929 and p < 0.001, 95% CI 0.019 to 0.867, respectively). The ankle joint diameter at the contralateral side was significantly higher in CFA 10.0 group in these specified days compared to CFA 5.0 (p < 0.001, 95% CI 0.044 to 1.283) and CFA 7.5 (p < 0.05, 95% CI -0.077 to 0.84) groups (Fig 7b).

### Inflammatory cells infiltration at the CFA-inoculated hind paw in chronic polyarthritic rats

**Fig 8:**
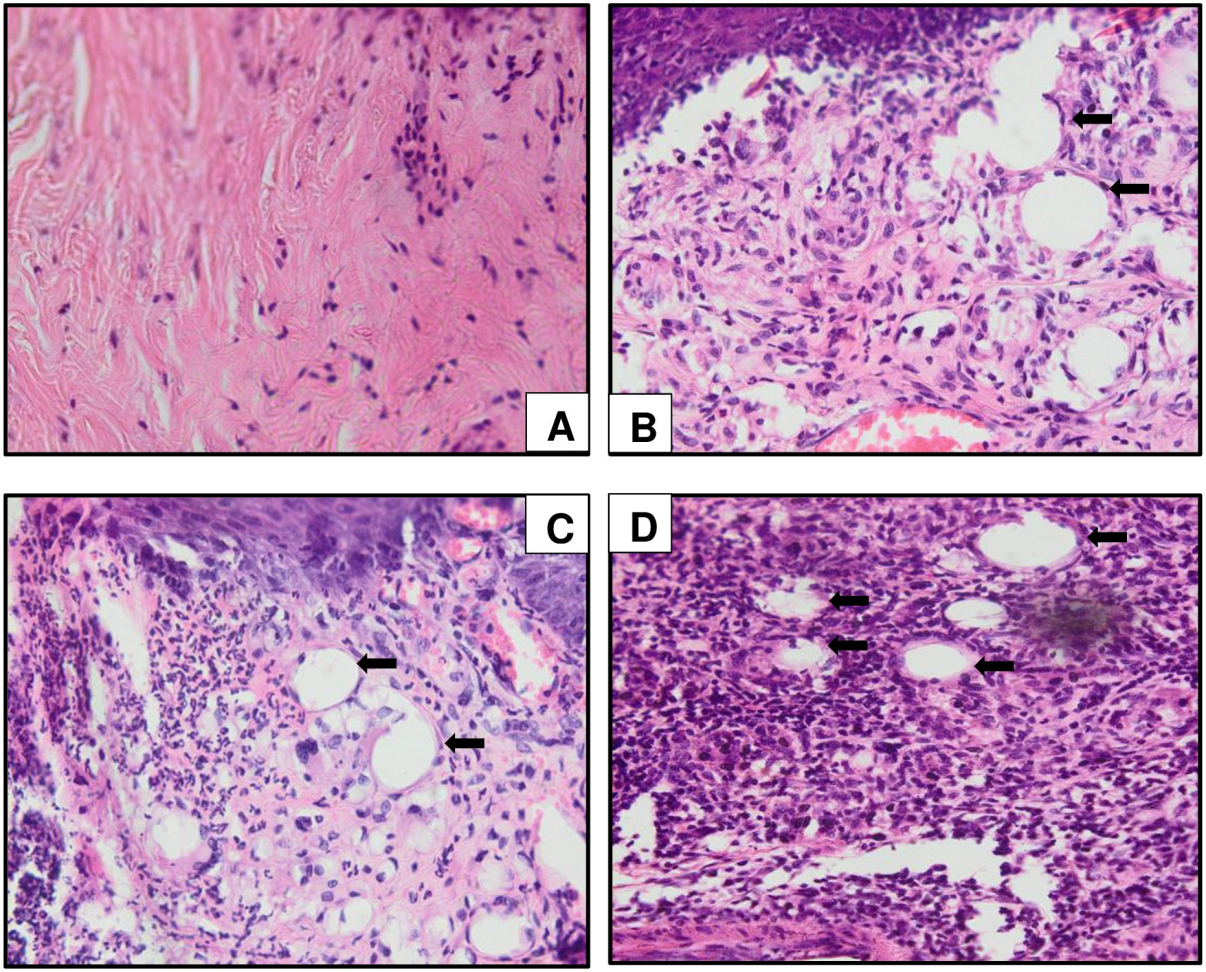
Histological sections of the CFA-inoculated hind paw tissues in (A) control, (B) CFA 5.0, (C) CFA 7.5 and (D) CFA 10.0 subjected to haematoxylin and eosin (H&E) staining at 400x magnification. The control group demonstrated no significant lesion appearance in the mineral oil-injected hind paw. Meanwhile, extensive inflammatory cells infiltration was observed in CFA 10.0 group, (D) mild and moderate level of inflammatory cells infiltration were detected in CFA 5.0 (B) and CFA 7.5 (C), respectively. The arrow indicates the extensive inflammatory cells infiltration at the injected hind paw tissue.

It is revealed that the infiltration of the inflammatory cells in the paw tissue of the chronic polyarthritic rats in dose-dependent manner as the presence of inflammatory cells infiltration including white blood cells was the mildest in CFA 5.0 group, moderate in CFA 7.5 group and the highest in the CFA 10.0 group. The rats inoculated with 10 mg/mL of CFA showed the most severe inflammation with the extensive cells infiltration (Fig 8).

### Histological changes in the ipsilateral ankle joint of CFA-induced polyarthritic rat

**Fig 9:**
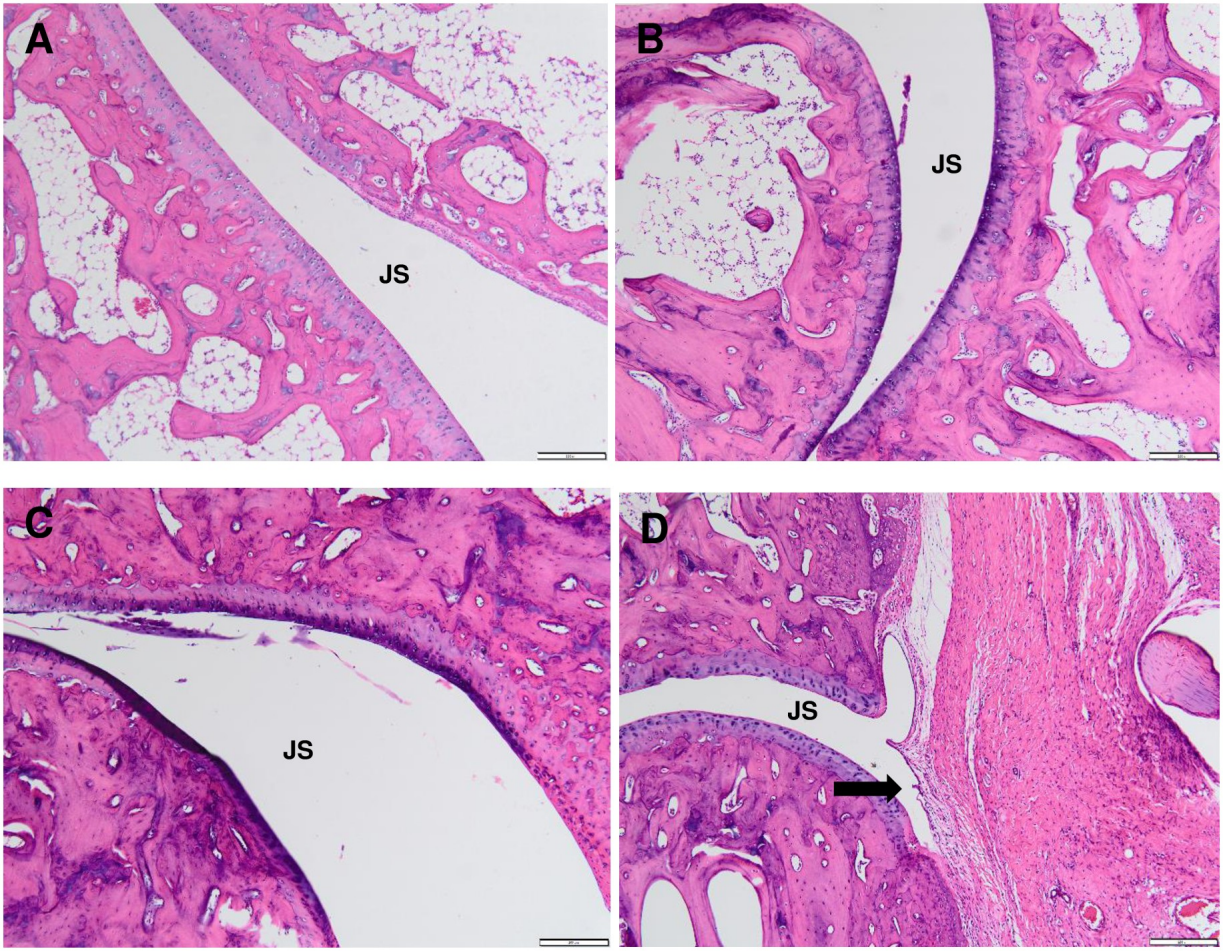
Representative histological sections of ipsilateral ankle joint by haematoxylin and eosin (H&E) staining at 100x magnification in control (A), polyarthritic rat inoculated with CFA at 5.0 mg/mL (CFA 5.0) (B), 7.5 mg/mL (CFA 7.5) (C) and 10.0 mg/mL (CFA 10.0) (D). (A) In the control group, smooth articulation of the joint cartilage surface, regular joint space (JS) with the normal connective tissue of the synovial membrane were observed. Meanwhile, ipsilateral ankle joint of rats in CFA 5.0 (B) and CFA 7.5 (C) groups showed mild penetration of inflammatory cells into the synovial cavity, indicating suppressed joint pathology and less soft tissue swelling. Additionally, the synovial hyperplasia with extensive inflammatory cells infiltration (as shown by the purplish stains), bone resorption and severe cartilage destruction indicating the narrowing of the joint with inflammatory cell infiltration into the synovial cavity (arrow) were found in the ipsilateral ankle joints of CFA 10.0 group.

**Fig 10:**
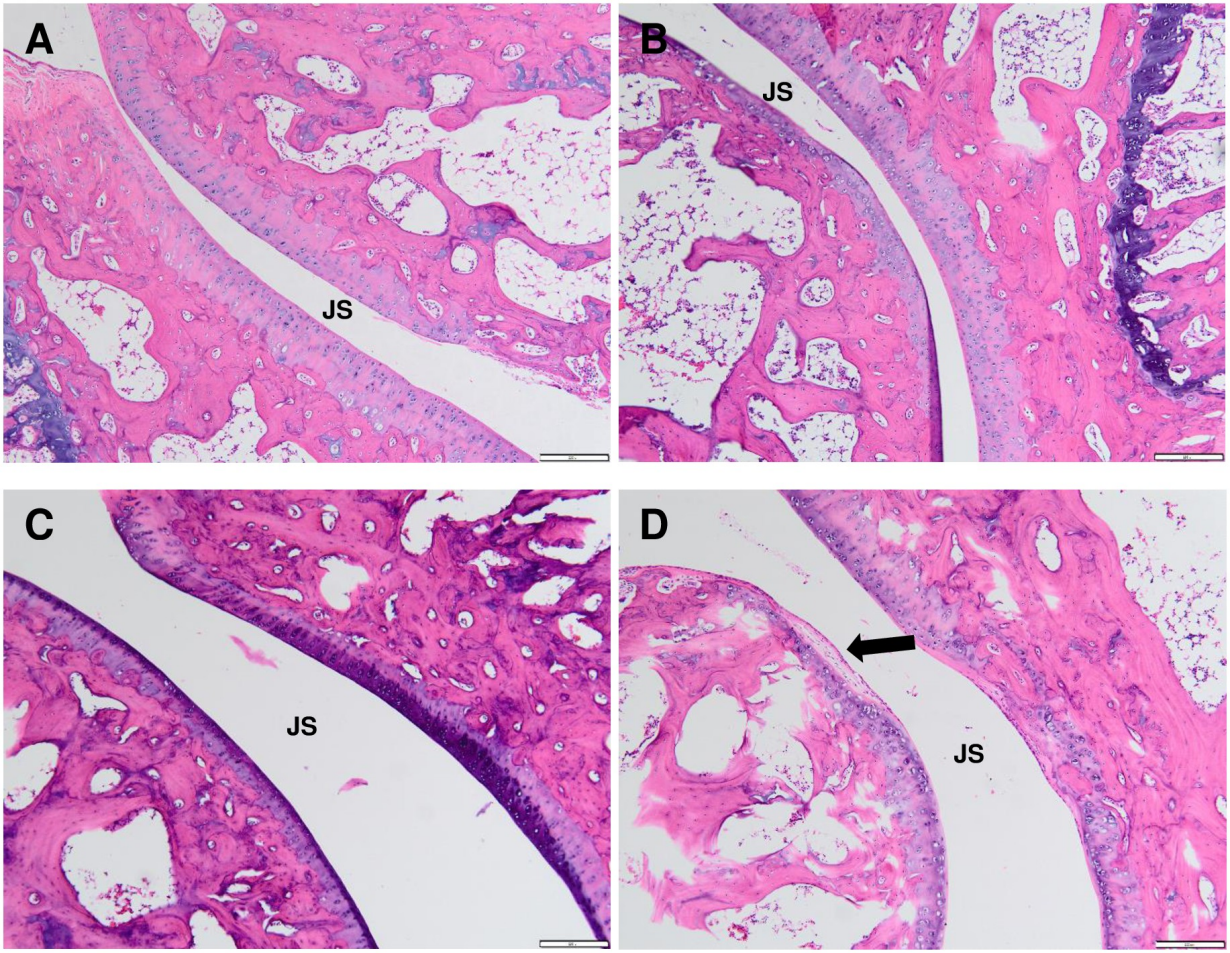
Histological sections of contralateral ankle joint by haematoxylin and eosin (H&E) staining at 40x magnification in control (A), polyarthritic rat inoculated with CFA at 5.0 mg/mL (CFA 5.0) (B), 7.5 mg/mL (CFA 7.5) (C) and 10.0 mg/mL (CFA 10.0) (D). (A) Like the ipsilateral ankle joint, the control rat exhibited normal joint cartilage structure, regular joint space with the normal connective tissue of the synovial membrane. CFA 5.0 group did not demonstrate prominent inflammatory cells penetration in the regular joint space. Meanwhile, the inflammatory cells infiltration was identified between the joint space (arrow) of the CFA 7.5 (C) and CFA 10.0 (D) groups, although it was not as extensive as the ipsilateral ankle joint.

The histopathology analysis as evaluated by the H&E staining demonstrated the extensive inflammatory cells infiltration in the synovial membrane especially in the ankle joint of CFA 10.0 group which was not found in the control group. The synovial hyperplasia was also prominent in CFA 10.0 group compared to the groups inoculated at lower and moderate dosage (CFA 5.0 and CFA 7.5 groups) (Fig 9). There was also the appearance of inflammatory cells infiltration identified at the contralateral ankle joint in CFA 7.5 and CFA 10.0 groups although the it was not as extensive as in the ipsilateral ankle joint (Fig 10).

### Hematological analysis

The full blood count measurements revealed significant differences between the groups for total white blood cells (WBCs, F_3,20_ = 3.271, p < 0.05), monocytes (F_3,20_ = 3.887, p < 0.05), eosinophils (F_3,20_ = 4.617, p < 0.05), lymphocytes (F_3,20_ = 14.723, p < 0.001), platelet counts (F_3,20_ = 24.414, p < 0.001), haematocrit (F_3,20_ = 11.434, p < 0.01), red cell distribution width (RDW, F_3,20_ = 6.764, p < 0.01) and polymorphs (F_3,20_ = 22.182, p < 0.001). No significant effects were detected between the groups for red blood cell count (RBCs), mean corpuscular volume (MCV), mean corpuscular haemoglobin (MCH) and mean corpuscular haemoglobin concentration (MCHC) (Table 1). The CFA 7.5 group showed significant alteration in the monocyte and lymphocyte counts, haematocrit, haemoglobin and polymorphs compared to control group (p < 0.05). However, most of the haematological parameters investigated was significantly changed in the group inoculated with CFA at 10 mg/mL (CFA 10.0) compared to control group indicating the chronic inflammation occurs in this group (Table 1).

**Table 1:** Full blood count in the groups.

Haematological parameters	Control	CFA 5.0	CFA 7.5	CFA 10.0
RBCs (10^6^/mcL)	7.77 ± 0.103	7.15 ± 0.2	7.15 ± 0.164	7.2 ± 0.219
Total WBCs (10^3^/μL)	5.333 ± 0.103	8.1 ± 3.834*	5.85 ± 0.3	8.55 ± 1.807*
Differential counts (%):				
i) **Monocytes**	7.03 ± 0.051	10.8 ± 0.876*	10 ± 1.095*	32 ± 28.48***
ii) **Eosinophils**	1.03 ± 0.051	0.00 ± 0.00	0.0 ± 0.00	7.0 ± 7.67***
iii) **Lymphocytes**	77.92 ± 0.491	60.0 ± 21.81	59.5 ± 4.93*	21.0 ± 11.9***
iv) **Basophils**	0.00 ± 0.00	0.00 ± 0.00	0.00 ± 0.00	0.00 ± 0.00
Haemoglobin (g/dL)	163.0 ± 0.00	146.0 ± 5.477*	141.5 ± 2.739*	142.0 ± 4.93*
Platelets (billion/L)	653.5 ± 0.837	653.0 ± 135.8*	751.8 ± 0.408*	1061.5 ± 135.3**
MCV (fL)	55.0 ± 0.537	55.5 ± 2.236	54.5 ± 2.168	53.5 ± 0.447
MCH (pg)	21.5 ± 0.447	20.5 ± 0.447	20.75 ± 1.084	20.0 ± 0.013
Haematocrit (L)	0.42 ± 0.00	0.4 ± 0.00	0.377 ± 0.004*	0.385 ± 0.013
MCHC (g/L)	390 ± 10.00	370 ± 11.1	380 ± 19.3	370 ± 12.8
RDW (%)	14.95 ± 0.134	15.45 ± 0.313	15.9 ± 0.563	15.95 ± 0.581*
Polymorphs (%)	19.0 ± 4.472	11.0 ± 0.321	30.5 ± 4.92*	44.67 ± 13.692**

*p < 0.05, **p < 0.01 and ***p < 0.001 significantly different compared to control group.

RBC = red blood cell count, WBCs = white blood cell counts, MCV = mean corpuscular volume, MCH = mean corpuscular haemoglobin, MCHC = mean corpuscular haemoglobin concentration and RDW = red cell distribution width.

The values are presented as mean ± standard deviation (S.D.) (n = 6).

## Discussion

The CFA-induced arthritic model was first developed by Stoerk et al. [56] and has been extensively modified for investigations on either acute or chronic, mono- or polyarthritis and inflammatory mechanisms. Various CFA doses were tested to establish arthritic rat models in earlier literature via subcutaneous, intradermal or intraarticular routes. However, few studies included the nociceptive and inflammatory effects in identifying the optimal CFA dose for developing a chronic polyarthritic rat model mimicking RA in humans. In this study, rats were inoculated with high dosages of CFA, ranging from 5.0 mg/mL to 10.0 mg/mL; several alterations were observed in the animals’ body weight, spontaneous activities, development of tactile allodynia, thermal hyperalgesia, joints inflammatory oedema, joint and hind paw morphological structures and haematological indices.

According to Costa et al. [7], a clinical disease that develops after 15–30 days following intradermal inoculation of 5.0 mg/mL CFA at the base of the tail in an arthritic rat is considered to be acute. On the other hand, Butler et al. [33] stated that the stable arthritic progression in rats for over six weeks could be classified as chronic. In the present study, the rats were inoculated with 5 to 10 mg/mL of CFA at their hind paws, potentially producing more chronic systemic effects in the rat’s body than Costa et al. [7]. In addition, Wauben et al. [57] reported that the CFA dose ranging from 1.0 to 5.0 mg/mL might produce less severe effects, while 10 mg/mL CFA may result in more severe effects, which aligned with the findings of the current study. Since this study aimed to develop an arthritic rat model mimicking RA, the outcome must be polyarthritic rather than monoarthritic rats, involving more than one affected joint. Moreover, inoculating CFA at the hind paw may produce little systemic disturbance compared to the inoculation of CFA at the base of the tail that resulted in more severe systemic changes and poor rat health [33]. Therefore, this study may guide researchers in selecting the optimal CFA dosage to be inoculated intradermally at the hind paw to establish a RA rat model.

Body weight change is one of the evaluation criteria in disease progression. The rats that were inoculated with a high dose of CFA (10 mg/mL) showed substantial body weight loss, but it was not significantly different from the lower and moderate doses groups. Nevertheless, it indicates the active disease development contributed to the changes in body weight [58, 59] since the total food intake was similar in both arthritic rats and normal rats. On top of that, lower weight gain found in the CFA 10.0 group was consistent with the fact that RA is correlated with the loss of lean body mass, referred to as rheumatoid cachexia (the loss of muscle mass and strength due to RA development) [15]. It is believed that the reduced body weight gain is implicated by the inflammation from the arthritic condition resulting in impaired nutrients absorption in the small intestine of the rat [59, 60]. Furthermore, the abnormal neuroendocrine response to the prolonged inflammation in RA was characterised by the increased catabolic hormones (i.e. glucocorticoids) and the decreased anabolic factors secretion (i.e. insulin-like growth factor-1) with elevated inflammatory cytokines, leading to hypermetabolism and a marked reduction in arthritic rat body mass [61].

The rats inoculated with CFA at all doses demonstrated a substantial increase in spontaneous activities indicated by walking difficulties with abnormal gait (i.e. limping to crawling in animals with high disability). Similarly, Simjee et al. [62] reported the gradual decline of walking and standing paw pressures in the rats inoculated with CFA, especially in the CFA 10.0 group. However, it remains unclear how the mild to moderate CFA inoculation led to the spontaneous activities fluctuation especially walking paw pressure, but it could be related to the inflammatory reactions within the experimental period. Thus, it was postulated that the mobility impediments could be an effect of joints pain, loss of functional balance or muscle loss [63]. Besides, Hayer et al. [64] demonstrated a progressive decline in the mobility of arthritic rat, suggested by gait patterns in the later phase of arthritis. Furthermore, the change in mobility may imply acute inflammatory processes following the adjuvant induction since several disease-modifying anti-rheumatoid drug treatments have been reported to improve the mobility of arthritic rats [62].

Besides, the present study showed that the paw withdrawal threshold significantly decreased at the ipsilateral and contralateral hind paws, especially in CFA 7.5 and CFA 10.0 groups, indicating tactile allodynia development following the CFA inoculation [65]. Among all the CFA doses, the inoculation of CFA at 10 mg/mL showed the best tactile allodynia progression in the chronic polyarthritic rat. On top of that, the rats inoculated with 7.5 and 10.0 mg/mL CFA showed a significant gradual decrease of thermal withdrawal threshold, which was a sign of thermal hyperalgesia [65]. These observations implied that the chronic polyarthritic model showed neuropathic-like symptoms, commonly found in RA patients of more than five years [66]. Moreover, Gomes et al. [40] suggested that hyperalgesia and allodynia in arthritis pathogenesis are the results of the extensive penetration of macrophages, lymphocytes, fibroblasts, and leukocytes into the joints where the inflammatory mechanism is responsible for several cytokines, prostaglandins, and proteolytic enzymes. These inflammatory mediators may have sensitised the peripheral nociceptors by inducing the phosphorylation of ligand-gated channels such as voltage-gated sodium channels, altering membrane properties, enhanced firing of action potential and heightened the sensitivity to thermal or mechanical (tactile) stimuli [67]. Besides peripheral sensitisation, central sensitisation may also reduce the nociceptive neuron’s threshold to fire the action potentials leading to hyperalgesia [67, 68].

Meanwhile, joint oedema at both ipsilateral and contralateral ankle joints strongly suggests the development of chronic polyarthritis in the rat model that could mimic RA in humans. Likewise, Mahdi et al. [39] reported dose-dependent joints oedema formation in their rat model, as shown in the present study. Out of all the administered dosages, rats treated with 10 mg/mL CFA demonstrated a consistent and gradual increase in circumference and diameter of both ankle joints throughout the experiment without fluctuations, proving the dosage efficacy in establishing chronic polyarthritis in a rat model. Furthermore, the arthritic symptoms were evident with hind paw swelling and reddening within 3–4 days following CFA inoculation, signs of acute inflammation followed by chronic inflammation [15, 42]. Furthermore, Szekanecz et al. [21] demonstrated that CFA inoculation contributed to joints oedema followed by swelling of ankle tarsus and dorsal area on day 11 due to the neutrophil infiltration and synovial lining proliferation, which correlates with the histological findings of the ankle joints structures in the present study. Interestingly, extensive inflammatory cell infiltration was detected in the hind paw tissue of CFA 10.0 rats compared to other treatment groups. These extensive histological changes may be the effect of hyper-responsiveness of activated macrophages at the affected site, leading to aberrant destructive outcomes [69]. Besides oedema formation, the cartilage degeneration and substantial infiltration of the inflammatory exudates into the articular surface also led to pannus formation [70]. These changes, which were also evident in previous studies, are the probable cause of the prominent histopathological changes in the CFA 10.0 group.

Based on the full blood count profile, there was a significant increase in total white blood cells, especially monocyte, eosinophil and lymphocyte levels in CFA 10.0 group compared to normal rats, indicating the prominent systematic inflammatory reactions in the arthritic rats. These results actually confirmed the systemic occurrence of chronic inflammation in this group, along with the presence of inflammatory cells infiltration at the hind paw and ankle joints and the chronic inflammatory oedema at the ankle joints in this study. Despite the lack of changes in RBC counts, the haemoglobin level in all CFA groups significantly declined, indicating the anaemic condition in the polyarthritic rat, resembling the iron deficiency anaemia in RA patients as previously reported by Mahdi et al. [39] and Paval et al. [71]. The anaemic condition in the arthritic rat may be attributed to the impaired iron storage in the reticuloendothelial system following arthritic development [72]. Meanwhile, the increased platelet counts in the CFA groups were dose-dependent, similar to the study by Naz et al. [72]. Therefore, the increased platelet count in the polyarthritic rat could be a consequence of the immune system stimulation towards the attenuated *M*. *butyricum* antigens in the animal’s body.

## Conclusion

In summary, CFA inoculation at 10 mg/mL in the rats’ hind paw was the optimal dose that closely mimics the nociceptive and inflammatory complications shown in human RA patients. Since its effects were the most distinct and consistent in terms of pain behaviour and inflammatory parameters, this dosage could be the most reliable and convincing in inducing RA in a rat model. Further investigations, including the presence of rheumatoid factor, C-reactive protein in the blood, nociceptive and inflammatory markers in several related organs, should be conducted in future studies to confirm the similarities of this model to RA in humans.

## Abbreviations

ANOVA: analysis of variance
CFA: complete Freund’s adjuvant
EDTA: ethylenediaminetetraacetic acid
ENA-78: epithelial-neutrophil activating peptide-78
ESR: erythrocyte sedimentation rate
IFA: incomplete Freund’s adjuvant
IL-1β: interleukin-1β
MCH: mean corpuscular haemoglobin
MCHC: mean corpuscular haemoglobin concentration
MCP-1: monocyte-chemoattractant protein-1
MCV: mean corpuscular volume
MIP-1α: macrophage-inflammatory protein-1α
RA: rheumatoid arthritis
RBC: red blood cells
RDW: red cell distribution width
SD: standard deviation
SEM: standard error of mean
SPSS: statistical package for the social sciences
TNF-α: tumour necrosis factor-α
WBCs: white blood cells

## References

[1] Kim JM, Kim HY. Pathogenesis of rheumatoid arthritis. J Korean Med Assoc. 2010; 53(10):853–61

[2] Barbour Kamil E, Helmick Charles G, Boring Michael, Brady Teresa J. Vital signs: Prevalence of doctor-diagnosed arthritis and arthritis-attributable activity limitation—United States, 2013–2015. MMWR Morb Mortal Wkly Rep. 2017; 66(9): 246–53. doi: 10.15585/mmwr.mm6609e1 28278145 PMC5687192

[3] van Vollenhoven Ronald F. Sex differences in rheumatoid arthritis: more than meets the eye… BMC Med. 2009; 7:12. doi: 10.1186/1741-7015-7-12 19331649 PMC2670321

[4] Ollier William ER, Harrison Beverley, Symmons Deborah. What is the natural history of rheumatoid arthritis? Best Pract Res Cl Rh. 2001; 15(1):27–48. doi: 10.1053/berh.2000.0124 11358413

[5] Allan Gibofsky. Epidemiology, pathophysiology, and diagnosis of rheumatoid arthritis: A synopsis. Am J Manag Care. 2014; 20(7 Suppl):S128–135. 25180621

[6] Hootman Jennifer M, Helmick Charles G, Barbour Kamil E, Theis Kristina A, Boring Michael A. Updated Projected Prevalence of Self-Reported Doctor-Diagnosed Arthritis and Arthritis-Attributable Activity Limitation Among US Adults, 2015–2040. Arthritis Rheumatol. 2016; 68(7):1582–1587. doi: 10.1002/art.39692 27015600 PMC6059375

[7] De Costa Mauricio Castro, De Sutter Paul, Gybels Jan, Van Hees Johan. Adjuvant-induced arthritis in rats: A possible animal model of chronic pain. Pain. 1981; 10:173–85. doi: 10.1016/0304-3959(81)90193-7 7267134

[8] Shivanand Pandey. Various techniques for the evaluation of anti arthrtic activity in animal models. J Adv Pharm Technol. 2010; 1(2):164–71. PMC3255441 22247842

[9] Jeffery Rachel C. Clinical features of rheumatoid arthritis. Medicine. 2014; 42(5):231–236.

[10] Grötsch B, Bozec A, Schett G. In vivo models of rheumatoid arthritis. In: Idris AI (editor). Bone Research Protocols. New York: Humana Press; 2019. pp. 269–80. 10.1007/978-1-4939-8997-3_14 30729470

[11] Asquith Darren L, Miller Ashley M, McInnes Iain B, Liew Foo Y. Animal models of rheumatoid arthritis. Eur J Immunol. 2009; 39(8):2040–2044. doi: 10.1002/eji.200939578 19672892

[12] Bernard Calvino, Crepon-Bernard Marie-Odile, Le Bars Daniel. Parallel clinical and behavioural studies of adjuvant-induced arthritis in the rat: Possible relationship with ‘chronic pain’. Behav Brain Res. 1987; 24(1):11–29. doi: 10.1016/0166-4328(87)90032-5 3580112

[13] Qiang Guo, Yuxiang Wang, Dan Xu, Johannes Nossent, Pavlos Nathan J Xu Jiake. Rheumatoid arthritis: Pathological mechanisms and modern pharmacologic therapies. Bome Res. 2018; 19:146. doi: 10.1038/s41413-018-0016-9 29736302 PMC5920070

[14] Fischer Bradford D, Adeyemo Adeshina, O’Leary Michael E, Bottaro Andrea. Animal models of rheumatoid pain: experimental systems and insights. Arthritis Res Ther. 2017. doi: 10.1186/s13075-017-1361-6 28666464 PMC5493070

[15] Cinzia Nasuti, Laura Bordoni, Donatella Fedeli, Rosita Gabbianelli. Effect of Nigella sativa Oil in a Rat Model of Adjuvant-Induced Arthritis. Multidisciplinary Digital Institute Proceedings. 2019; 11:16.

[16] Laste G, Souza IC, Santos VS, Caumo W, Torres IL. Histopathological changes in three variations of Wistar Rat adjuvant-induced arthritis model. Int J Pharmaceutical Res Schol. 2014; 3:780–790. doi: 10.13140/2.1.3260.5443

[17] Ke Ren, Ronald Dubner. Inflammatory models of pain and hyperalgesia. Institute for Lab Anim Res. 1999; 40(13):111–118. doi: 10.1093/ilar.40.3.111 11406689

[18] Neugebauer V, Han JS, Adwanikar H, Fu Y, Ji G. Techniques for assessing knee joint pain in arthritis. Mol Pain. 2007; 3:1744–8069. doi: 10.1186/1744-8069-3-8 17391515 PMC1851005

[19] Shasha Luo, He Li, Jingjing Liu, Xiaoqian Xie, Zhijie Wan, Yale Wang, et al. Andrographolide ameliorates oxidative stress, inflammation and histological outcome in complete Freund’s adjuvant-induced arthritis. Chem Biol Interact. 2020; 319:1–9. 10.1016/j.cbi.2020.108984 32061742

[20] Pearson C, Wood FD. Studies of polyarthritis and other lesions in rats by injection of mycobacterial adjuvant. I. General clinical and pathological characteristics and some modifying factors. Arthritis Rheum. 1959; 2(5):440–59.

[21] Szekanecz Z, Halloran MM, Volin MV, Woods JM, Strieter RM, Haines GK III, et al. Temporal expression of inflammatory cytokines and chemokines in rat adjuvant-induced arthritis. Arthritis Rheum. 2000; 43(6):1266–77. doi: 10.1002/1529-0131(200006)43:6&lt;1266::AID-ANR9&gt;3.0.CO;2-P 10857785

[22] Suke Sanvidhan G, Negi Harsh, Mediratta PK, Banerjee BD, Sharma KK. Anti-arthritic and anti-inflammatory activity of combined pioglitazone and prednisolone on adjuvant-induced arthritis. Eur J Pharmacol. 2013; 718:57–62. doi: 10.1016/j.ejphar.2013.09.019 24075936

[23] Alfons Biliau, Patrick Matthys. Modes of action of Freund’s adjuvants in experimental models of autoimmune diseases. J Leukoc Biol. 2001; 70:849–860. 11739546

[24] Hirayasu Kai, KKazuko Shibuya, Yinan Wang, Hirotaka Kameta, Tomie Kameyama, Satoko Tahara-Hanaoka, et al. Critical role of M. tuberculosis for dendritic cell maturation to induce collagen-induced arthritis in H-2b background of C57BL/6 mice. Immunology. 2006; 118:233–39. doi: 10.1111/j.1365-2567.2006.02361.x 16771858 PMC1782291

[25] Nguemnang Stephanie Flore Djuichou, Tsafack Eric Gonzal, Mbiantcha Marius, Gilbert Ateufack, Atsamo Albert Donatien, Nana William Yousseu, et al. *In vitro* anti-inflammatory and *in vivo* anti-arthritic activities of aqueous and ethanolic extracts of *Dissotis thollonii* cogn. (melastomataceae) in rats. Evid Based Complem Altern Med. 2019; doi: 10.1155/2019/3612481 31827550 PMC6881768

[26] Alessandra Zingoni, Rosa Molfetta, Cinzia Fionda, Alessandra Soriani, Rosella Paolini, Marco Cippitelli, et al. NKG2D and its ligands:“one for all, all for one”. Front Immunol. 2018; 9:476. doi: 10.3389/fimmu.2018.00476 29662484 PMC5890157

[27] Knight B, Katz DR, Isenberg DA, Ibrahim MA, Le Page S, Hutchings P, et al. Induction of adjuvant arthritis in mice. Clin Exp Immunol. 1992; 90(3):459–65. doi: 10.1111/j.1365-2249.1992.tb05868.x 1458683 PMC1554568

[28] Zhu CZ, Bannon AW, Joshi SK. Complete Freund’s adjuvant-induced reduction of exploratory activity in a novel environment as an objective nociceptive endpoint for sub-acute inflammatory pain model in rats. Eur J Pain. 2015; 19(10):1527–36. doi: 10.1002/ejp.686 25731687

[29] Chang Jennie CC, Diveley Jocelyn P, Savary Jay R, Jensen Fred C. Adjuvant activity of incomplete Freund’s adjuvant. Adv Drug Deliv Rev. 1998; 32(3):173–186. doi: 10.1016/s0169-409x(98)00009-x 10837643

[30] Juliana De Souza Apostólico, Victoria Alves Santos Lunardelli, Fernanda Caroline Coirada, Silvia Beatriz Boscardin, Daniela Santoro Rosa. Adjuvants: classification, modus operandi, and licensing. J Immunol Res. 2016. doi: 10.1155/2016/1459394 27274998 PMC4870346

[31] Rainsford KD. Adjuvant polyarthritis in rats: is this a satisfactory model for screening anti-arthritic drugs. Agents Actions. 1982; 12:452–458. doi: 10.1007/BF01965926 6758549

[32] Penny Hawkins, Rachel Armstrong, Tania Boden, Paul Garside, Katherine Knight, Elliot Lilley, et al. Applying refinement to the use of mice and rats in rheumatoid arthritis research. Inflammopharmacology. 2015; 23:131–50. doi: 10.1007/s10787-015-0241-4 26168847 PMC4508365

[33] Butler Stephen H, Godefroy Francoise, Besson Jean-Marie, Weil-Fugazza Jeanne. A limited arthritic model for chronic pain studies in the rat. Pain. 1992; 48(1):73–81. doi: 10.1016/0304-3959(92)90133-V 1738577

[34] Grubb BD, Birrell GJ, McQueen DS, Iggo A. The role of PGE2 in the sensitization of mechanoreceptors in normal and inflammed ankle joints of the rat. Exp Brain Res. 1991; 84:383–92. doi: 10.1007/BF00231460 2065745

[35] Donaldson Lucy F, Seckl Jonathan R, McQueen Daniel S. A discrete adjuvant-induced monoarthritis in the rat: Effects of adjuvant dose. J Neurosci Methods. 1993; 49(1–2): 5–10. doi: 10.1016/0165-0270(93)90103-x 8271831

[36] Cook Charles D, Moore Kathryn I. Effects of sex, hindpaw injection site and stimulus modality on nociceptive sensitivity in arthritic rats. Physiol Behav. 2006; 87(3): 552–62. doi: 10.1016/j.physbeh.2005.12.005 16455114

[37] Yuanyuan Wu, Xinmiao Yao, Yongliang Jiang, Xiaofen He, Xiaomei Shao, Junying Du, et al. Pain aversion and anxiety-like behaviour occur at different times during the course of chronic inflammatory pain in rats. J Pain Res. 2017; 10: 2585. doi: 10.2147/JPR.S139679 29158690 PMC5683785

[38] Xiuying Zhang, Yanfeng Dang, Hanyu Dong, Wen Zhang, Fang Li. Investigation of the effect of phlomisoside F on complete Freund’s adjuvant-induced arthritis. Exp Ther Med. 2016; 13:710–716. doi: 10.3892/etm.2016.3995 28352356 PMC5348710

[39] Mahdi Harith Jamel, Khan Nurzalina Abdul Karim, Asmawi Mohd Zaini, Mahmud Roziahanim, Murugaiyah Vikneswaran. In vivo anti-arthritic and anti-nociceptive effects of ethanol extract of Moringa oleifera leaves on complete Freund’s adjuvant (CFA)-induced arthritis in rats. Integr Med Res. 2018; 7(1):85–94. doi: 10.1016/j.imr.2017.11.002 29629295 PMC5884001

[40] Gomes Raquel Pinheiro, Bressan Elisangela, da Silva Tatiane Morgana, da Silva Gevaerd Monica, Tonussi Carlos Rogério, Domenech Susana Cristina. Standardization of an experimental model suitable for studies on the effects of exercise on arthritis. *Einstein (São Paulo)*. 2013; 11(1):76–82. doi: 10.1590/s1679-45082013000100014 23579748 PMC4872972

[41] Naeini Reza Sharif, Cahill Catherine M, Ribeiro-Da-Silva Alfredo, Ménard Henri A, Henry James L. Remodelling of spinal nociceptive mechanisms in an animal model of monoarthritis. Eur J Neurosci. 2005; 22(8):2005–2015. doi: 10.1111/j.1460-9568.2005.04382.x 16262639

[42] Laste G, Ripoll Rozisky J, De Macedo IC, Souza Dos Santos V, Custódio De Souza IC, Caumo W, et al. Spinal cord brain-derived neurotrophic factor levels increase after dexamethasone treatment in male rats with chronic inflammation. NeuroImmunoModulation. 2013; 20:119–25. doi: 10.1159/000345995 23328256

[43] Coderre Terrence J, Wall Patrick D. Ankle joint urate arthritis (AJUA) in rats: an alternative animal model of arthritis to that produced by Freund’s adjuvant. Pain. 1987; 28(3):379–93. doi: 10.1016/0304-3959(87)90072-8 3574965

[44] Zulazmi Nurul Atiqah, Gopalsamy Banulata, Omar Farouk Ahmad Akira, Sulaiman Mohd Roslan, Bharatham B Hemabarathy, Perimal Enoch Kumar. Antiallodynic and antihyperalgesic effects of zerumbone on a mouse model of chronic constriction injury-induced neuropathic pain. Fitoterapia. 2015; 105:215–21. doi: 10.1016/j.fitote.2015.07.011 26205045

[45] Deuis Jennifer R, Dvorakova Lucie S, Vetter Irina. Methods used to evaluate pain behaviors in rodents. Front Mol Neurosci. 2017; doi: 10.3389/fnmol.2017.00284 28932184 PMC5592204

[46] Tawfik Vivianne L, Nutile-McMenemy Nancy, LaCroix-Fralish Michael L, DeLeo Joyce A. Efficacy of propentofylline, a glial modulating agent, on existing mechanical allodynia following peripheral nerve injury. Brain Behav Immun. 2007; 21(2):238–246. doi: 10.1016/j.bbi.2006.07.001 16949251

[47] Steinar Hunskaar, Odd-Geir Berge, Kjell Hole. A modified hot-plate test sensitivie to mild analgesics. Behav Brain Res. 1986; 21(2):101–8. doi: 10.1016/0166-4328(86)90088-4 3755945

[48] Langford Dale J, Mogil Jeffrey S. Pain Testing in the Laboratory Mouse. 2^nd^ Ed. In: Fish RE, Brown MJ, Karas AZ. Anesthesia and Analgesia in Laboratory Animals. Academic Press: 2008; 549–560.

[49] Arnau Hervera, Sergi Leánez, Roberto Motterlini, Olga Pol. Treatment with carbon monoxide-releasing molecules and an HO-1 inducer enhances the effects and expression of μ-opioid receptors during neuropathic pain. Anesthesiology. 2013; 118:1180–97. doi: 10.1097/ALN.0b013e318286d085 23358127

[50] Escandell José M, Recio Carmen M, Máñez Salvador, Giner Rosa M, Cerdá-Nicolás Miguel, Ríos José Luis. Cucurbitacin R reduces the inflammation and bone damage associated with adjuvant arthritis in Lewis rats by suppression of tumor necrosis factor-α in T lymphocytes and macrophages. J Pharmacol Exp Ther. 2007; 320(2):581–90. doi: 10.1124/jpet.106.107003 17065367

[51] Ali Eman A I, Barakat Bassan M, Hassan Ranya. Antioxidant and angiostatic effect of spirulina platensis suspension in complete freund’s adjuvant-Induced arthritis in rats. *PLoS One*. 2015; 10:1–13. doi: 10.1371/journal.pone.0121523 25853428 PMC4390336

[52] Chillingworth Naomi L, Donaldson Lucy F. Characterisation of a Freund’s complete adjuvant-induced model of chronic arthritis in mice. J Neurosci Methods. 2003; 128(1–2):45–52. doi: 10.1016/s0165-0270(03)00147-x 12948547

[53] Likun Cui, Wenzhe Zhu, Zhijie Yang, Xiyuan Song, Cui Xu, Ziwei Cui, et al. Evidence of anti-inflammatory activity of Schizandrin A in animal models of acute inflammation. Naunyn-Schmiedeberg’s Arch Pharmacol. 2020; 393:2221–2229. 32076762 10.1007/s00210-020-01837-x

[54] Ahmed Khader Syed Zameer, Ahmed Sidhra Syed Zameer, Thangakumar Arunachalam, Krishnaveni Radhakrishnan. Therapeutic effect of Parmotrema tinctorum against complete Freund’s adjuvant-induced arthritis in rats and identification of novel Isophthalic ester derivative. Biomed Pharmacother. 2019; 112:108646. doi: 10.1016/j.biopha.2019.108646 30970506

[55] Sakai A, Hirano T, Okazaki R, Okimoto N, Tanaka K, Nakamura T. Large-dose ascorbic acid administration suppresses the development of arthritis in adjuvant-injected rats. Arch Orthop Trauma Surg. 1999; 119:121–126. doi: 10.1007/s004020050374 10392503

[56] Stoerk HT, Bielinski T, Budzilovich T. Chronic polyarthritis in rats injected with spleen in adjuvants. Am J Pathol. 1954; 30(3):616.

[57] Wauben Marca H, Wagenaar-Hilbers Josée P, van Eden Willem. Chapter 13—Adjuvant arthritis. In: Cohen Irun R, Miller Ariel (editors). Autoimmune disease models. United States: Academic Press. 1994; 211–216.

[58] Naik SR, Wala SM. Arthritis, a complex connective and synovial joint destructive autoimmune diseases: animal models of arthritis with varied etiopathology and its significance. J Postgrad Med. 2014; 60(3):309–317. doi: 10.4103/0022-3859.138799 25121375

[59] Xin Cui, Ruijing Wang, Peimin Bian, Qingke Wu, Seshadri Vidya Devanatha Desikan, Lun Liu. Evaluation of antiarthritic activity of nimbolide against Freund’s adjuvant induced arthritis in rats. Artif Cell Nanomed B. 2019; 47(1):3391–3398. 10.1080/21691401.2019.1649269 31394949

[60] Patil Kalpana S, Suryavanshi Jayaprakash. Effect of Celastrus paniculatus Willd. seed on adjuvant induced arthritis in Rats. Pharmacogn Mag. 2007; 3(11):177–181.

[61] Miriam Granado, Martín Ana I, Angeles Villanúa M, López-Calderón Asunción. Experimental arthritis inhibits the insulin-like growth factor-I axiz and induces muscle wasting through cyclooxygenase-2 activation. Am J Physiol Endocrinol Metab. 2007; 292(6):E1656–1665. doi: 10.1152/ajpendo.00502.2006 17284570

[62] Usman Simjee Shabana, Huma Jawed, Javeria Quadri, Arshad Saeed Sheikh. Quantitative gait analysis as a method to assess mechanical hyperalgesia modulated by disease-modifying antirhumatoid drugs in the adjuvant-induced arthritic rat. Arthritis Res Ther. 2007; 9:R91. doi: 10.1186/ar2290 17848187 PMC2212551

[63] Claude Mossiat, Davy laroche, Clément Prati, Thierry Pozzo, Céline Demougeot, Christine Marie. Association between arthritis score at the onset of the disease and long-term locomotor outcome in adjuvant-induced arthritis in rat. Arthritis Res Ther. 2015; 17:184. doi: 10.1186/s13075-015-0700-8 26183428 PMC4506462

[64] Silvia Hayer, Gregor Bauer, Martin Willburger, Katharina Sinn, Farideh Alasti, Roberto Plasenzotti, et al. Cartilage damage and bone erosion are more prominent determinants of functional impairment in longstanding experimental arthritis than synovial inflammation. Dis Model Mech. 2016; 9:1329–1338. doi: 10.1242/dmm.025460 27638666 PMC5117225

[65] Yukinori Nagakura, Masamichi Okada, Atsuyuki Kohara, Tetsuo Kiso, Takashi Toya, Akihiko Iwai, et al. Allodynia and hyperalgesia in adjuvant-induced arthritic rats: Time course of progression and efficacy of analgesics. J Pharmacol Exp Ther. 2003; 306(2):490–497. doi: 10.1124/jpet.103.050781 12730275

[66] Koop SN, Ten Klooster PM, Vonkeman HE, Steunebrink LM, van de Laar MA. Neuropathic-like pain features and cross-sectional associations in rheumatoid arthritis. Ann Rheum Dis. 2015; 17(Suppl 2):237. doi: 10.1186/s13075-015-0761-8 26335941 PMC4558794

[67] Pinho-Ribeiro Felipe A, Verri Waldiceu A Jr, Chiu Isaac M. Nociceptor sensory neuron-immune interactions in pain and inflammation. Trends Immunol. 2017; 38(1):5–19. doi: 10.1016/j.it.2016.10.001 27793571 PMC5205568

[68] Borsook D. Chapter 223 Neuropathic pain. In: Samuels MA, Feske SK, editors. Office Practice of Neurology. 2^nd^ Edn. Churchill Livingstone. 2003; 1402–1407.

[69] Zhao J, Zhao M, Yu C, Zhang X, Liu J, Cheng X, et al. Multifunctional folate receptor-targeting and pH-responsive nanocarriers loaded with methotrexate for treatment of rheumatoid arthritis. Int J Nanomed. 2017; 12:6735–6746. doi: 10.2147/IJN.S140992 28932117 PMC5600269

[70] Meera Sumanth, Anusha Swetha S. Elucidation of mechanism of anti-arthriticaction of Arthosansar- a polyherbal formulation. Indian J Tradit Knowl. 2012; 11(4):704–713

[71] Paval J, Kaitheri SK, Potu BK, Govindan S, Kumar RS, Narayanan SN, et al. Anti-arthritic potential of the plant *Justicia gendarussa* Burm F. Clinics. 2009; 64(4):357–562. doi: 10.1590/s1807-59322009000400015 19488595 PMC2694464

[72] Rabiya Naz, Zaheer Ahmed, Muhammad Shahzad, Arham Shabbir, Faiza Kamal. Amelioration of rheumatoid arthritis by *Anarcadium occidentale* via inhibition of collagenase and lysosomal enzymes. Evid Based Complem Altern Med. 2020; doi: 10.1155/2020/8869484 33224258 PMC7669349

